# Estimating the temporal and spatial risk of bluetongue related to the incursion of infected vectors into Switzerland

**DOI:** 10.1186/1746-6148-4-42

**Published:** 2008-10-15

**Authors:** V Racloz, G Venter, C Griot, KDC Stärk

**Affiliations:** 1 Monitoring, Swiss Federal Veterinary Office, Bern, Switzerland; 2 Division of Entomology, Agricultural Research Council, Onderstepoort Veterinary Institute, South Africa; 3 Institute of Virology and Immunoprophylaxis, Mittelhäusern, Switzerland; 4 Infection and Immunity Group, Royal Veterinary College, London, UK

## Abstract

**Background:**

The design of veterinary and public health surveillance systems has been improved by the ability to combine Geographical Information Systems (GIS), mathematical models and up to date epidemiological knowledge. In Switzerland, an early warning system was developed for detecting the incursion of the bluetongue disease virus (BT) and to monitor the frequency of its vectors. Based on data generated by this surveillance system, GIS and transmission models were used in order to determine suitable seasonal vector habitat locations and risk periods for a larger and more targeted surveillance program.

**Results:**

Combined thematic maps of temperature, humidity and altitude were created to visualize the association with *Culicoides *vector habitat locations. Additional monthly maps of estimated basic reproduction number transmission rates (R_0_) were created in order to highlight areas of Switzerland prone to higher BT outbreaks in relation to both vector activity and transmission levels. The maps revealed several foci of higher risk areas, especially in northern parts of Switzerland, suitable for both vector presence and vector activity for 2006.

Results showed a variation of R_0 _values comparing 2005 and 2006 yet suggested that Switzerland was at risk of an outbreak of BT, especially if the incursion arrived in a suitable vector activity period. Since the time of conducting these analyses, this suitability has proved to be the case with the recent outbreaks of BT in northern Switzerland.

**Conclusion:**

Our results stress the importance of environmental factors and their effect on the dynamics of a vector-borne disease. In this case, results of this model were used as input parameters for creating a national targeted surveillance program tailored to both the spatial and the temporal aspect of the disease and its vectors. In this manner, financial and logistic resources can be used in an optimal way through seasonally and geographically adjusted surveillance efforts. This model can serve as a tool for other vector-borne diseases including human zoonotic vectors which are likely to spread into Europe.

## Background

Bluetongue disease virus (BT) is a vector-borne, infectious but non-contagious animal pathogen. This emerging disease affects all ruminants and has been responsible for an unprecedented continuing European epidemic which has been occurring for the past decade [1]. Belonging to the Orbivirus genus and Reoviridae family, there are currently 24 recognized serotypes transmitted globally by a multitude of *Culicoides *midge species, each with their own habitat preferences and geographical distribution albeit with some overlapping occurrence. Several serotypes, mostly affecting sheep, have been circulating in the Balkan and Mediterranean areas since the late 90's, which could be predicted by the advance of its Old World vector *C. imicola*. Yet, an outbreak of BT serotype 8 (BTV-8) in 2006, which was last recorded in the African and the Caribbean region [2], suddenly occurred in Northern Europe, an area previously free of BT infection [3]. Preceding this event, outbreaks had been reported on a regular seasonal basis in southern Europe, mainly the Mediterranean region involving several serotypes namely BTV -1, -2, -4, -6, -9 and -16 [1].

At the time of writing, the first occurrence of BTV-8 in Switzerland was reported in northern Switzerland in the canton of Basel-City in late October 2006. This was shortly followed by cases in the canton of Solothurn, and Basel-Land respectively. Further cases of BTV-8 were consecutively detected in Basel-Land and Solothurn. In January 2007, a surprising case was discovered in the southern canton of Valais. In the first outbreaks of BT ever recorded in Switzerland dating from October 2006 to February 2007, a total of 12 cattle and two goats tested positive for BTV-8 originating from seven different farms. Although no firm conclusion have yet arisen as to the cause of these cases, wind direction patterns along with temperature records of the affected areas suggest likely intrusion of infected vectors originating from the surrounding BT-reporting areas.

Due to the dynamics of the pathogen, combined with the fact that the geographical limits of other vector-borne diseases are also expanding, Switzerland conducted a nation-wide survey in 2003 to determine the status of BT disease and the presence of its vectors [4]. Additionally since 2005 [5], studies conducted have recorded the presence of the following potential BT vectors: *C. obsoletus *(Meigen), *C. pulicaris, C. scoticus *and *C. dewulfi*, species which have all been implicated in BT outbreaks in several countries located in Europe and the Balkan areas [3,6,7]

**Figure 1:**
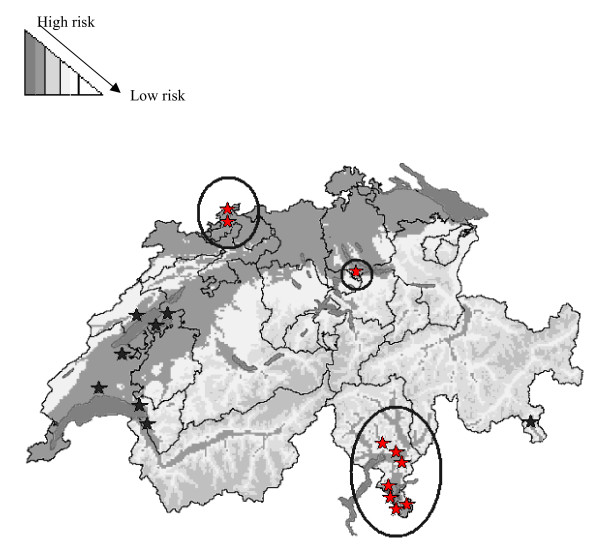
**Location of sentinel herds for BT early detection from 2004–2007 in Switzerland**. Background colour scheme represents risk zones in terms of general *Culicoides *habitat suitability for 2006. Black stars are location of sentinel herds involved in serological sampling, red stars indicate location of sentinel herds undergoing both serological and entomological sampling. Circles include herds which contributed data for this study.

Although this resulted in proving freedom of infection, the presence of vector species competent of transmitting BT were found to be abundant in various areas of the country. This in turn prompted the establishment of a sentinel herd surveillance system through serological and entomological monitoring, focusing on certain areas of the country (Fig. 1) [8,9]. Due to the nature of vector-borne diseases, and the fact that BT was still absent in Switzerland, risk based sampling was implemented [9]. This risk-based design involved identifying geographical areas which matched habitat criteria for the presence, survival and establishment of a vector species [5,8], which were identified through GIS mapping techniques [10]. However, these maps were limited to annual analysis, and did not differentiate different levels of vector activity throughout the year, nor did they consider rainfall and snow data in certain months. Hence a more informative and detailed mapping method alongside a mathematical model was created.

The aim of this study was to combine GIS maps of data collected in the field with maps of predicted basic reproduction number estimates for potential BT outbreaks in Switzerland, in order to explore the spatial and temporal regions more prone to 1) the establishment of important vector populations, and 2) enabling the spread of the disease due to the nature of geographical and climatic features.

## Results

### Temperature variability and R_0 _calculations

**Figure 2:**
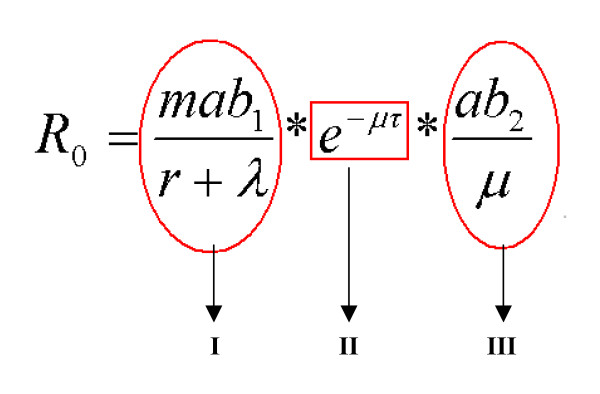
**R_0 _equation**. Equation for calculating the Basic Reproductive number, circled I, II, and III to represent the different components.

**Figure 3:**
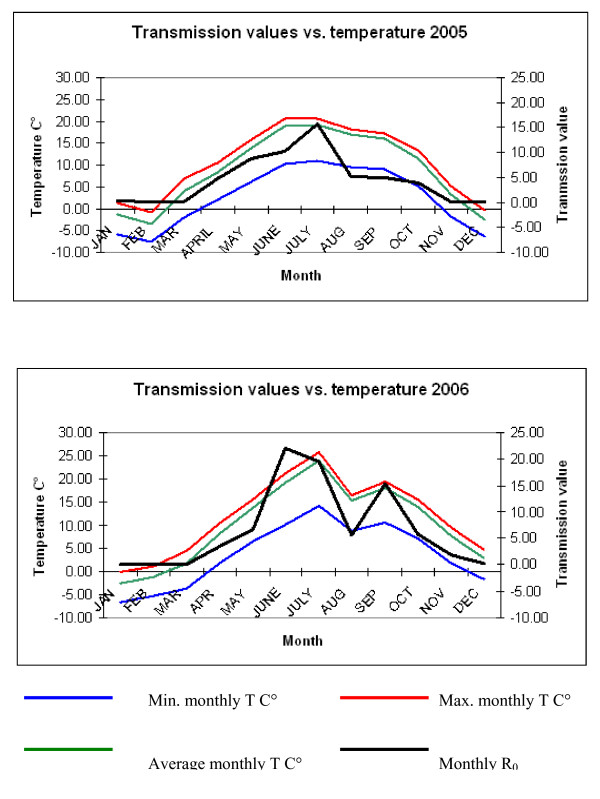
**a) Monthly R_0 _values plotted versus monthly temperature for Switzerland, 2005**. Calculated R_0 _values for 2005 in Switzerland (secondary y-axis) along with monthly mean, minimum and maximum temperatures (primary y-axis). **b) Monthly R_0 _values plotted versus monthly temperature for Switzerland, 2006**. Calculated R_0 _values for 2006 in Switzerland (secondary y-axis) along with monthly mean, minimum and maximum temperatures (primary y-axis)

**Figure 4:**
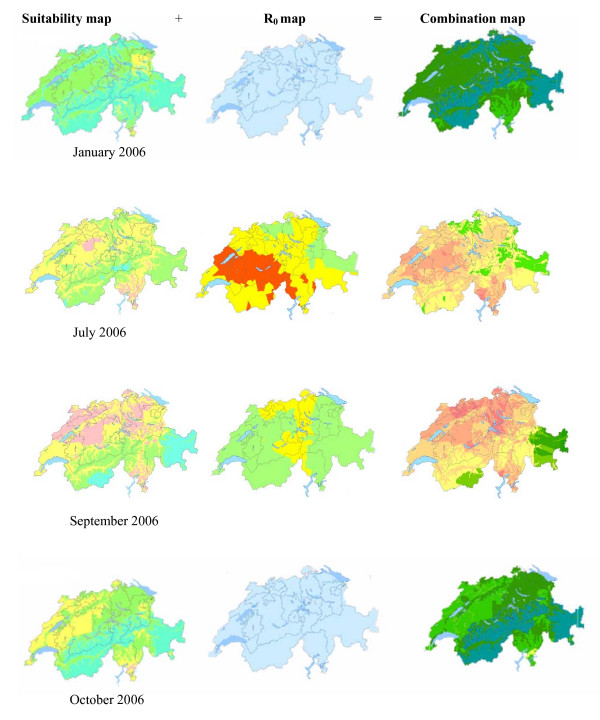
**Suitability maps of BT occurrence in Switzerland, 2006**. Suitability maps (left margin) which were added to R_0 _maps (middle margin) to create final combination maps (right margin) Selected months of January, July, September and October are shown.

The basic reproduction number R_0 _(Fig. 2), was defined as the 'expected number of secondary cases that would arise from a typical primary case in a susceptible population' [11], as proposed for other vector-borne diseases such as Malaria [12], West Nile fever [13], African Horse sickness [14], as well as recently for BT [15]. The monthly R_0 _values along with temperature records between the years 2005 and 2006 were considerably different. Figure 3a shows that the R_0 _peak for 2005 occurred during July with a maximum value of 15, as compared to 2006 (Fig 3b) which had two R_0 _peaks occurring in June and September reaching R_0 _values of 22 and 16 respectively. Results showed that R_0 _followed a pattern similar to recorded monthly temperatures (Fig. 3a and 3b). June was the warmest month in 2005, with a mean temperature of 20.7°C as compared to 2006, where the warmest month of July recorded a mean monthly temperature of 25.7°C (Fig. 3b). Similarly, the month of February in 2005 recorded the lowest mean minimum temperature of -7.6°C whilst in 2006 a minimum of -6.5°C was recorded in January. The two most considerable differences observed which affect vector biology and activity rates, were the higher recorded temperatures during the summer months, accompanied by a milder winter period for the year of 2006 as compared to 2005. Monthly variations were also observed in both years following seasonal patterns as shown in the suitability maps in Figure 4 (data only for 2006).

### GIS mapping

The combined results of the suitability maps and the R_0 _maps for the months of January, July, September and October of 2006 are shown in Figure 4. The suitability maps (Fig 4, left) highlight various localized microclimates occurring in Switzerland and confirm the role of the Alps in separating the northern and southern parts of the country. The maps (Fig. 4, left) showed that some areas remain suitable for *Culicoides *survival throughout the year despite colder temperatures, agreeing with virus over-wintering theories. In contrast though, the R_0 _maps showed negligible risk of BT spread in the colder months of 2006 (Fig. 4, middle), due to low vector activity rates. However when combined, the risk maps (Fig 4, right) showed that the risk of an outbreak and spread of BT in Switzerland was not negligible even in the colder seasons due to the continued suitability for vector survival in certain areas, noting that *Culicoides *vectors have also been recorded within farms where local temperatures (personal communication) would be efficient for virus transmission.

The difference between the vector suitability maps and the calculated transmission values are most distinct when comparing the maps for July and September of 2006. July is less suitable for vector reproduction due to the high temperatures recorded in that month which could, among other factors, affect larval death rates through desiccation, yet R_0 _values are higher than in the month of September, due to the shortened extrinsic incubation period. On the other hand, the maps for September show more suitable areas for vector activity through the effect of less extreme temperatures.

## Discussion

Our results showed that the months of July and September 2006 were the most suitable period in Switzerland for vector presence in terms of climatic conditions (suitability maps), while the highest R_0 _value indicating vector activity and therefore spread, occurred in June. This is an interesting finding in relation to the BT incursion and subsequent outbreak in Northern Europe, which was first detected in August 2006, possibly indicating similar conditions in the affected countries, such as The Netherlands, Belgium and Germany. The R_0 _values estimated in this study were used as a risk indicator levels for vector activity in different geographic areas (Fig. 4 middle) in terms of BT transmission rates. They were then combined with maps indicating areas with predicted higher vector suitability likelihood (Fig. 4 left). The actual cases of BT correspond to the maps generated for 2006, yet due to the data originating from this year, cannot be replicated for future outbreaks of BT. Similarly to the mapping methods for climate, R_0 _values were classified into 4 risk levels, signifying most rapid spread of disease in areas presenting higher range of R_0 _values.

The basic reproduction number (R_0_) for vector-borne diseases is a more complex number to calculate due to the influence of seasonal fluctuations [16], local climate and environmental features as well as the abundance of breeding sites available near hosts which affect vector dynamics [14]. The transmission rates of the disease will also change depending on temperature factors affecting vector to host or host to vector interaction, along with the extrinsic incubation time, biting rates and vector mortality rates [15]. In this study, vector density numbers along with temperature values were used from field data collected throughout the project in order to produce R_0 _values specific to the areas and time frame studied. The values which were most affected in terms of location and time period were those of vector density, vector death rates and extrinsic incubation rates. These were mainly affected by differences in temperature and humidity levels which in turn are very specific to the various microclimates present in Switzerland targeted in the sampling. Concerning the effect of host availability on transmission rates, since data was taken from sentinel farms where minimal transhumance activity took place, cattle density was considered as stable, with a Swiss average size of 30 cattle per farm.

A recent study by Gubbins et al. [15] assessed the risk of BT in the United Kingdom using the basic reproduction number R_0_. Similarly to their findings, the R_0 _values were highest when temperatures were between 15°C–25°C. Whilst the mentioned study took into account both the ratio of vectors to cattle and sheep in the formula, our study only considered the relation of BTV-8 in cattle farms and not sheep due to much larger cattle population of larger ruminants present in Switzerland. Additionally in financial terms, the cattle industry and more specifically the milk sector is an important part of the Swiss economy .

For Switzerland, the peak of R_0 _can be explained by the fact that the largest amount of *Culicoides *midges in 2006 were recorded in June, probably due to optimal breeding and hatching conditions as May was a mild and humid month. July held the record for maximum temperature, which leads to higher vector activity and successful virus transmission. However high temperatures also increase vector mortality rate and thereby lower R_0 _values for this month. Due to the very different meteorological patterns in the past three years, we would therefore expect that maps and R_0 _for 2007 (comparable to conditions in 2003) to be quite different compared to 2005 and 2006 in terms of increased vector activity and BT transmission rates. .

Affected by the initial high temperatures recorded in July 2006, transmission values as well as vector density decreased significantly following a notably milder August. In terms of the Northern European BTV8 outbreak data, this would match reports stating that the maximum number of cases occurred in October which would originate from a high number of vectors present a few weeks previously. Due to the nature of *Culicoides *development, it has been suggested that cases occur at a time lag of circa four weeks from peak vector density periods, which corroborate the evidence from the trapping data and the transmission values [17]. As seen in The Netherlands which recorded high temperatures in July, this climatic event could have primed the vectors in terms of their competence and capacity levels, translating in an effective transmission period, as observed in many other affected countries [17]. As mentioned in a study conducted by Murray [18], rapid changes in climatic conditions can affect vector population age structure, along with vector density and alter transmission rates of disease.

A limiting factor in our model is that vector density data resulted from trapping sites representing high risk areas for entomological surveillance, located in southern parts of Switzerland. In these locations, high numbers of vectors were expected. Therefore, the transmission values may be overestimated in some parts of the country, yet due to the targeted nature of setting up sentinel herds in high risk areas this limitation was acceptable in this scenario.

Another factor to consider is that only the dynamics between BTV-8 and the vectors belonging to the *Culicoides obsoletus *group were studied, and considering that a majority of the biological parameters used in calculating values for R_0 _were derived from the available literature, this should be taken into account when applying such methods to different countries, especially when micro-climates or other BT vectors are present. Outbreaks involving other BT serotypes and their dynamics in *Culicoides *vectors produce different R_0 _values [19]. They may also have a different preference for distinct geographic and climatic conditions. Such differences have been described for the behaviour of BTV-2 and *Culicoides imicola *in southern France [20], and the role of *C. imicola *in South Africa [21].

The recent cases of BT in Switzerland in the month of October 2007 occurred in the northern part of the country, where temperatures for the affected region were similar to those of 2006, and the number of cattle affected on the seven farms correspond to the R_0 _figures calculated for that area and time period. Out of a total of 608 susceptible cattle, 16 sheep and six goats, originating from the farms located in Basel-City, Solothurn, Basel-Land, Valais and Jura, BT was detected in a total of 12 cattle and 2 goats, with prevalence rates ranging in chronological terms of 10.3% to 0.6% in cattle and 33% in goats . The R_0 _values provided by the model were similar to the actual prevalence rates for the time of the year, with the exception of goats, which were not included in the study.

Our findings highlight the potential for establishing a flexible surveillance system taking into account environmental factors. In a targeted surveillance system, this could mean increased serological testing during a specific warmer period or in specific geographical areas. Given the scarcity of epidemiological data available for Switzerland concerning BT cases prior to this study, the creation of thematic and risk maps on a monthly and annual basis, illustrated the variability in the behaviour of vector borne diseases and the possible consequences of virus introduction. It also provided the basis for creating a surveillance system targeting higher BT-risk regions and months. In the case of Switzerland, the maps and R_0 _values were used as input parameters for the creation of a BT surveillance Scenario Tree model [25], with the aim of comparing the efficacy of alternative surveillance system designs. A risk-based surveillance program was implemented in July 2007 consisting of three surveillance system components; serological bulk milk testing of 200 sentinel herds located in areas considered of higher risk to BT occurrence, as well as clinical surveillance programs for cattle and sheep farmers throughout the country.

## Conclusion

GIS mapping techniques combined with statistical and mathematical models can help improve disease surveillance and control methods by providing a basis for targeting monitoring efforts. Targeted surveillance in this scenario meant focusing financial and monitoring efforts to pre-defined areas originating from the maps created in this study.

An advantage of GIS methods includes the ability to improve prediction maps once more comprehensive field data has been collected, and adjust surveillance efforts in a timely and accurate manner. Flexible surveillance programs should be used in order to attribute financial and human resources to high-risk areas considering temporal and spatial factors.

In this study, maps were used to highlight areas in Switzerland which presented higher likely risk for vector habitat, which incorporated climatic elements, (Figure 4. left margin), combined with maps highlighting different vector activity levels based on R_0 _values for BT (Figure 4. middle column), in order to produce a set of final combination maps representing areas in Switzerland most likely to have outbreaks of BT due to vector presence in relation to vector activity levels on a monthly basis for 2006. As mentioned, the different risk zone distribution originating from the final combination maps were used as input parameters in a Scenario Tree model to aid in decision making processes concerning BT surveillance in Switzerland .

Approaches using GIS maps, and the combination of spatial analysis and mathematical modelling as predictive tools have been used in other countries such as Italy, Spain, and France concerning BT and its vectors [22-24], and stress the advantages of using different technological methods in supporting surveillance efforts, as well as for other purposes aiding in the improvement of health and prevention of disease both in public and veterinary terms.

## Methods

### R_0 _calculations

One aim of the study was to determine the potential consequence of a bluetongue outbreak, using the basic reproductive number (R_0_) and incorporating local climate data as well as Swiss *Culicoides *abundance information. The latter information deriving from entomological data collected using Onderstepoort blacklight traps in sampling sites for the years 2005 and 2006[5]. Vector abundance data along with local temperature information were collected from ten sentinel herd sites located in various sites throughout Switzerland (Fig. 1). Insects were collected with a trapping frequency of twice per month, each with two successive nights per sampling session. A total of 63 and 46 samples for 2005 and 2006 were collected, with 27, 256 and 43,527 *Culicoides *spp being identified respectively. Minimum and maximum temperature during trapping, insect abundance and diversity, host species present and altitude for each trap location were also recorded and monthly means for temperature were obtained from the Swiss Meteorological Office. Based on previous Malaria [12] and West Nile models [13], as well as a recent publication on BT R_0 _[15], hypothetical transmission values representing new BT cases per month for both years were estimated using the following equation (Figure 2). Values and symbols used in the equation are explained in Table 1.

**Table 1:** Parameters and values used for calculation of R_0_

**Symbol**	**Unit**	**Biological meaning**	**Values**	**Reference**
m	midge/trapping night	Vector density (average)	1–584	[5]
a	bite/day	Vector biting rate	0.25	[15]
b_1_	successful bites/midge	Transmission from vector to cattle	0.01	[28]
b_2_	%infectious bite/infected host	Transmission from cattle to vector	0.9	[26]
r	cattle/day	Recovery rate of cattle	0.04	[17]
λ	cattle/day	Cattle death rate	0.00008	[17]
e		2.718	2.718	Universal value
μ	vector/day	Vector death rate	0.1–0.6	[29]
*τ*	Days	Extrinsic incubation period	4–28	[28]

Symbols and their biological meaning used to calculate BT transmission values for Switzerland

In terms of vector-borne diseases, the basic reproduction number (R_0_), is defined as the number of new infections that would result from the introduction of a single infectious vector specimen into a completely susceptible/naive population of hosts [13]. The R_0_equation is made of three components (Fig. 2). Component (I) consists of the following symbols: vector density (m), derived from *Culicoides *catches in the national entomological surveillance, vector biting rates (a), transmission rates from vector to cattle (b_1_), cattle recovery rates (r) and cattle death rates (λ). This part of the equation involves the stage of vectors infecting their hosts. The second section (II) includes the extrinsic incubation period (*τ*), vector death rates (μ) and signifies the amount of time the virus is developing inside the vector taking into account the lifespan of the vector. Finally, the third component (III), which relates to the percentage of successful infectious bites per infected host, includes the vector biting rates (a), the transmission rates from cattle to vector (b_2_) and the vector death rates (μ).

Through the variation of extrinsic incubation periods relating to temperature and humidity levels, as well as the collected vector abundance data derived from field data, R_0 _values were estimated for each month for the years 2005 and 2006. The R_0 _values calculated were then categorised into 4 levels (high, medium-high, medium-low and low), symbolising vector activity levels in terms of BT virus transmission, for incorporation into GIS maps (Fig. 4. middle). Additionally, the R_0_values were plotted against monthly minimum, mean and maximum temperatures for the area where entomological trapping occurred (Fig. 3a and 3b).

### GIS mapping

Separate thematic maps were created using ArcGis (Version 8.3, Environmental Systems Research Institute, Inc.) for monthly mean temperature, altitude and humidity for the years of 2005 and 2006 using data from 50 meteorological measuring stations provided by the Swiss Meteorological Office, as previously described [10]. The aim was to create combined monthly vector suitability maps using these parameters to visualize the variation in potential risk areas during each season. Once monthly datasets were incorporated into the map, smoothing out was performed through kriging, apart from the altitude map which was derived from an elevation model. Suitability categories, based on *Culicoides obsoletus *group biology and habitat data [1,6,7,26-28], were used to reclassify the output values, in order to grade all monthly maps on a standard scale, as done for the R_0 _values mentioned above. The 'environmental envelope' of the *Obsoletus *group of *Culicoides *was concentrated upon in contrast to the classical Old World vector *C. imicola*, due the fact that the former is the most abundant group caught in the Swiss entomology surveillance program [9] and has been shown to transmit BT virus in other countries [3]. The maps were then layered together using the addition function in the raster map calculator which created a single combined suitability map for each month.

In a similar fashion, R_0 _values for each month were assigned to the 50 geographical coordinates based on the location of the meteorological stations, and kriging was performed in order to smooth out the data. The R_0 _values were divided into four categories, and reclassified to share a standardized scale for each month, as well as being comparable to the suitability map scales. The two sets of maps were then combined by adding the respective layers for each month through the raster map function to produce final combination maps incorporating both spatial and temporal factors (Fig. 4 right).

## Competing interests

The authors declare that they have no competing interests.

## Authors' contributions

VR and KS conceived the idea for the study. VR conducted all analytical work and wrote the manuscript. GV provided expert opinion on entomological aspects, CG provided expert advice on BT disease. All authors read, edited and approved the final version of the manuscript.
